# Supplementary material for: Understanding External Influences on Target Detection and Classification Using Camera Trap Images and Machine Learning
Source: Sensors (Basel). 2022 Jul 19;22(14):5386. doi: 10.3390/s22145386 (PMC9319727; doi:10.3390/s22145386)
Supplement: Supplementary file 1 [file sensors-22-05386-s001.zip › Supplemental Material.pdf]

Supplementary Material

Analyst-Daylight-Detection Probability (DP) Pre-Outlier Model

Table S1 Summary of the best fit data before outlier removal for the analyst-daylight-DP model, with vegetation type, occlusion, distance, colour and orientation towards the CT.

Variable	Estimate	95% Confidence intervals	P-value	Random effect (SD)
Fixed effects				
Intercept	181714.875024223	(4.376, 7545059276.243)	0.026	
Vegetation type				
Riverine forest	0.277	(0.247, 0.311)	<0.001	
Colour				
Green	0.781	(0.712, 0.857)	<0.001	
Distance				
5 meters	1675.092	(0.267, 10528679.554)	0.096	
10 meters	7.687	(4.505, 13.118)	<0.001	
15 meters	0.445	(0.369, 0.536)	<0.001	
20 meters	0.046	(0.038, 0.056)	<0.001	
25 meters	0.026	(0.021, 0.032)	<0.001	
30 meters	0.009	(0.007, 0.011)	<0.001	
Occlusion				
34-67%	0.001	(1.688×10^{-8}, 17.325)	0.155	
68-100%	0.000	(4.522×10^{-9}, 4.644)	0.095	

Orientation towards the CT				
Yes	1.356	(1.177, 1.562)	<0.001	
Random effect				
Experiment number				0.799

Analyst-Daylight-DP Post-Outlier Model

Table S2 Summary of the best fit data after outlier removal for the analyst-daylight-DP model, with vegetation type, occlusion, distance, colour and orientation towards the CT.

Variable	Estimate	95% Confidence intervals	P-value	Random effect (SD)
Fixed effects				
Intercept	2.047×10^{8}	(1.456×10^{5}, 2.880×10^{11})	<0.001	
Vegetation type				
Riverine forest	0.277	(0.247, 0.311)	<0.001	
Colour				
Green	0.781	(0.711, 0.856)	<0.001	
Distance				
10 meters	7.690	(4.502, 13.137)	<0.001	
15 meters	0.445	(0.369, 0.536)	<0.001	
20 meters	0.046	(0.038, 0.056)	<0.001	
25 meters	0.026	(0.021, 0.032)	<0.001	

30 meters	0.009	(0.007, 0.011)	<0.001	
Occlusion				
34-67%	4.800×10^{-7}	(3.394×10^{-10}, 6.789×10^{-4})	<0.001	
68-100%	1.287×10^{-7}	(9.082×10^{-11}, 1.825×10^{-4})	<0.001	
Orientation towards the CT				
Yes	1.356	(1.177, 1.562)	<0.001	
Random effect				
Experiment number				0.799

ML-Daylight-DP Pre-Outlier Model

Table S3 Summary of the best fit data before outlier removal for model ML-daylight-DP, with vegetation type, occlusion and distance.

Variable	Estimate	95% Confidence interval	P-value	Random effect (SD)
Fixed effect				
Intercept	321.893	(21.322, 4859.655)	<0.001	
Vegetation type				
Riverine forest	0.278	(0.231, 0.334)	<0.001	
Distance				
5 meters	3611.616	(0.001, 2.514×10^{10})	0.308	
10 meters	8.170	(4.089, 16.324)	<0.001	
15 meters	0.618	(0.479, 0.797)	<0.001	

20 meters	0.069	(0.053, 0.091)	<0.001	
25 meters	0.049	(0.037, 0.064)	<0.001	
30 meters	0.016	(0.012, 0.023)	<0.001	
Occlusion				
34-67%	0.143	(0.042, 0.484)	0.002	
68-100%	0.033	(0.010, 0.111)	<0.001	
Random effect				
Experiment number				0.806

ML-Daylight-DP Post-Outlier Model

Table S4 Summary of the best fit data after outlier removal for model ML-daylight-DP, with vegetation type, occlusion and distance.

Variable	Estimate	95% Confidence interval	P-value	Random effect (SD)
Fixed effect				
Intercept	321.893	(21.322, 4859.655)	<0.001	
Vegetation type				
Riverine forest	0.278	(0.231, 0.334)	<0.001	
Distance				
10 meters	8.176	(4.089, 16.345)	<0.001	
15 meters	0.618	(0.479, 0.797)	<0.001	
20 meters	0.069	(0.053, 0.091)	<0.001	

25 meters	0.049	(0.037, 0.064)	<0.001	
30 meters	0.016	(0.012, 0.023)	<0.001	
Occlusion				
34-67%	0.143	(0.042, 0.484)	0.002	
68-100%	0.033	(0.010, 0.111)	<0.001	
Random effect				
Experiment number				0.806

ML-Dusk-DP Pre-Outlier Model

Table S5 Summary of the best fit data before outlier removal for the ML-dusk-DP model, with occlusion and distance.

Variable	Estimate	95% Confidence interval	P-value	Random effect (SD)
Fixed effects				
Intercept	192.688	(29.664, 1251.645)	<0.001	
Distance				
10 meters	1.508	(1.232, 1.844)	<0.001	
15 meters	2.926	(2.418, 3.540)	<0.001	
20 meters	0.235	(0.175, 0.314)	<0.001	
25 meters	2.115×10^{-5}	(6.173×10^{-18}, 72444111.734)	0.465	
30 meters	2.115×10^{-5}	(6.173×10^{-18}, 72444111.734)	0.465	
Occlusion				

34-67%	0.038	(0.012, 0.120)	<0.001	
68-100%	0.000	(6.306×10^{-5}, 0.001)	<0.001	
Random effect				
Experiment number				0.714

ML-Dusk-DP Post-Outlier Model

Table S6 Summary of the best fit data after outlier removal for the ML-dusk-DP model, with occlusion and distance.

Variable	Estimate	95% Confidence interval	P-value	Random effect (SD)
Fixed effects				
Intercept	6.423	(3.275, 12.597)	<0.001	
Distance				
10 meters	0.852	(0.657, 1.104)	<0.001	
15 meters	1.497	(0.908, 4.760)	<0.001	
20 meters	0.605	(0.268, 0.942)	<0.001	
Occlusion				
34-67%	1.449	(1.027, 2.045)	<0.001	
68-100%	0.003	(0.002, 0.004)	<0.001	
Random effect				
Experiment number				0.714

ML-Dusk-Correct Classification (CC) Pre-Outlier Model

Table S7 Summary of the best fit data before outlier removal for the ML-dusk-CC model, with occlusion and distance.

Variable	Estimate	95% Confidence interval	P-value	Random effect (SD)
Fixed effects				
Intercept	1862.329	(92.222, 37607.935)	<0.001	
Distance				
10 meters	23891.701	(7.528×10^{-85}, 7.583×10^{92})	0.923	
15 meters	0.096	(0.012, 0.783)	0.029	
20 meters	0.000	(2.391×10^{-5}, 0.002)	<0.001	
25 meters	7.42×10^{-3}	(4.271×10^{-5}, 0.015)	<0.001	
30 meters	1.232×10^{-4}	(5.221×10^{-7}, 0.162)	<0.001	
Occlusion				
34-67%	1.732	(0.101, 29.578)	0.704	
68-100%	0.992	(0.060, 16.443)	0.995	
Random effect				
Experiment number				2.683

ML-Dusk-CC Post-Outlier Model

Table S8 Summary of the best fit data after outlier removal for the ML-dusk-CC model, with occlusion and distance.

Variable	Estimate	95% Confidence interval	P-value	Random effect (SD)
Fixed effects				

Intercept	422.749	(94.991, 744.651)	<0.001	
Distance				
15 meters	0.097	(0.013, 0.781)	0.005	
20 meters	0.002	(2.38×10^{-5}, 0.004)	<0.001	
25 meters	7.42×10^{-3}	(4.271×10^{-5}, 0.015)	<0.001	
30 meters	1.232×10^{-4}	(5.221×10^{-7}, 0.162)	<0.001	
Occlusion				
34-67%	0.092	(0.001, 2.510)	0.002	
68-100%	0.001	(1.227×10^{-2}, 0.129)	<0.001	
Random effect				
Experiment number				2.683
